# Resection and Abdominal Wall Reconstruction for Cesarean Scar Endometriosis

**DOI:** 10.1155/2022/7330013

**Published:** 2022-04-29

**Authors:** Kento Takaya, Hirokazu Shido, Shun Yamazaki

**Affiliations:** ^1^Department of Plastic and Reconstructive Surgery, Keio University School of Medicine, Tokyo, Japan; ^2^Yamato Municipal Hospital, Kanagawa, Yamato, Japan

## Abstract

**Introduction:**

Currently, there are few reports describing the use of reconstructive techniques in the treatment of cesarean scar endometriosis (CSE). Here, we report a case of CSE, a rare form of endometriosis caused by scars from obstetric and gynecological surgeries. *Case Report*. A 50-year-old woman became aware of a painful, deep scar mass in her lower abdomen during her menstrual period 10 years after her second cesarean section. This was diagnosed as CSE after the biopsy. Under general anesthesia, the mass, a portion of the rectus abdominis, and a 1 cm tumor-free margin were resected as a whole, and the abdominal wall was reconstructed with a soft artificial mesh.

**Results:**

No obvious recurrence or subjective symptoms were observed postoperatively or reported in the 1-year follow-up period. *Discussion*. Endometriosis appearing in a cesarean scar is rare; it is chiefly triggered by intraoperative mechanical implantation. In cases of surgical scar masses with a history of gynecological surgery and associated menstrual symptoms, this syndrome should be considered during diagnosis and treatment.

## 1. Introduction

Endometriosis is a benign gynecological disease characterized by the ectopic presence of active endometrial tissue outside the uterine cavity. Endometriosis occurs most commonly in the abdominal cavity, mainly in the ovaries, peritoneum, uterine ligament, and rectovaginal septum. Among cases of endometriosis occurring in the abdominal wall, those occurring in postcesarean scars are relatively rare, estimated to account for approximately 0.08% of all cases of endometriosis [1]. However, the incidence of this disease is estimated to be on the rise owing to the increase in cesarean sections worldwide [2]. Although many studies have described the treatment methods (including surgical resection and pharmacotherapy) and outcomes of endometriosis in postcesarean scars [1, 3], only a few reports describe the use of reconstructive techniques such as abdominal wall plasty and the flap technique, to prevent recurrence and herniation [4–6]. We report a case of endometriosis in a cesarean section scar that required partial abdominal wall reconstruction.

## 2. Case Presentation

**Figure 1:**
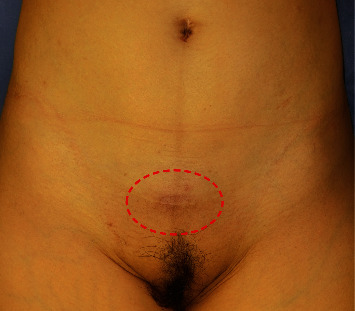
Findings on initial examination. A palpable subcutaneous mass is seen at the position surrounded by the red dashed line.

**Figure 2:**
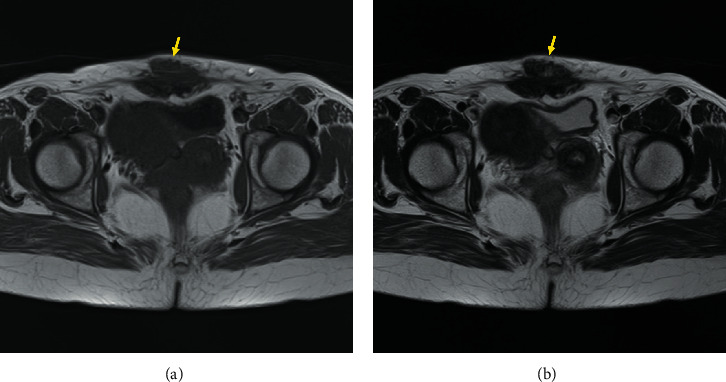
Preoperative MR images. (a) T1-weighted image. (b) T2-weighted image. A hypointense mass is observed within the rectus abdominis, as indicated by the yellow arrow. MR, magnetic resonance.

**Figure 3:**
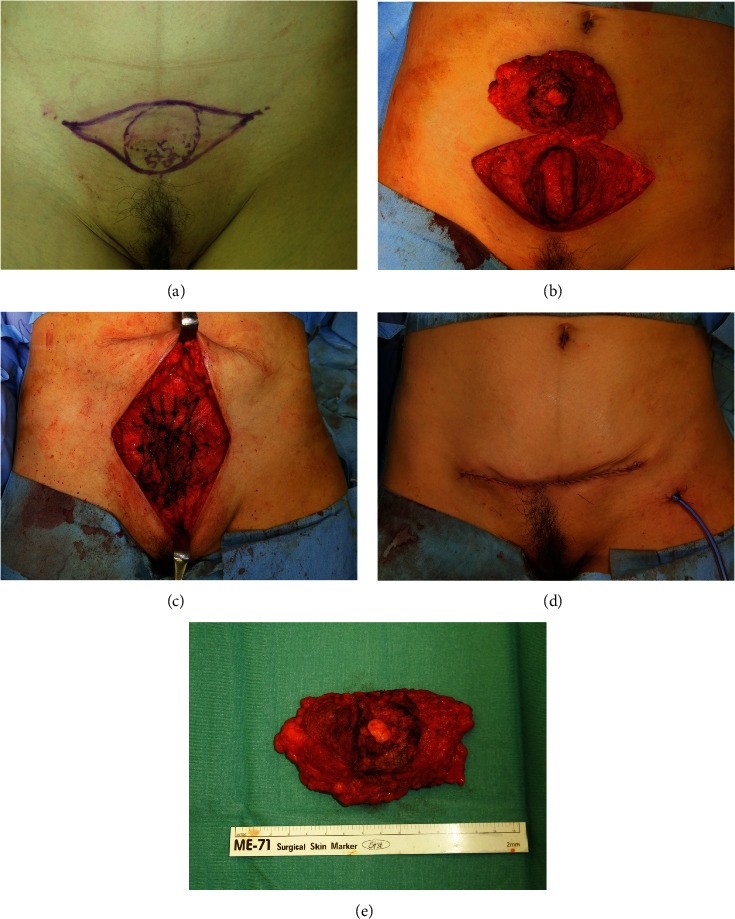
Intraoperative findings. (a) Preoperative design. (b) After tumor resection. (c) Abdominal wall reconstruction using a mesh. (d) Wound closure. (e) Removed specimen.

A 50-year-old woman presented with a painful lower abdominal mass that appeared during her menstrual cycle 10 years after her second cesarean section. The patient had a history of two cesarean sections, and no history of drinking, smoking, or taking medication. Magnetic resonance imaging (MRI) performed at the first hospital suggested a desmoid tumor; therefore, she was placed under observation. However, the size of the mass increased, and her subjective symptoms worsened; consequently, she visited the gynecology department at our hospital. The high levels of CA19-9 (152.21 U/mL) and CA125 (48.0 U/mL) in a blood test, in addition to her symptoms, led to the suspicion of endometriosis. We performed an excisional biopsy, which confirmed the diagnosis of endometriosis. Hormone therapy using oral luteinizing hormone was commenced but discontinued because of nausea. Surgery was planned for a radical cure. At the time of the visit, a mature scar with a transverse incision was found in the lower abdomen, and a golf ball-sized hard mass was palpable under the scar. The mass was partially adherent to the overlying skin and firmly adherent to the underlying rectus abdominis, and there was no skin redness or heat (Figure 1). MRI showed a substantial mass in the lower portion of the rectus abdominis, with slightly irregular borders and hypointensity on T1WI and T2WI (Figure 2). Surgery was performed under general anesthesia. After a skin incision along the scar tissue, a mass adherent to the anterior sheath of the rectus abdominis and partially infiltrating the muscle was identified. This mass was resected together with the rectus abdominis as a single mass, including a 1 cm margin. In addition, the posterior sheath of the rectus abdominis was partially resected, and the abdominal wall was reconstructed by implanting a soft artificial mesh (Figure 3).

**Figure 4:**
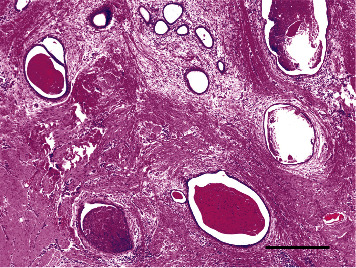
Pathologic observations. Endometrial tissue, hemosiderin, phagocytic histiocytes, and granulation tissue are observed. Bar = 500 *μ*m.

Histopathological examination of the excised specimen revealed endometrial tissue, hemosiderin, phagocytic histiocytes, and granulation tissue; hence, the patient was diagnosed with endometriosis (Figure 4). The patient was satisfied with the results of the surgery and did not report any apparent recurrence or complications, such as hematoma, infection, or hernia, during the 1-year follow-up period.

## 3. Discussion

Scar endometriosis is rarely reported in the gynecological literature and occurs in patients who have previously undergone obstetric and gynecological surgeries such as cesarean section, hysterectomy, perineal incision, and tubal ligation [7, 8]. Previous reports reviewed cases of CSE diagnosed from 1951 to 2006 and showed that the incidence of CSE was 0.08% [1]; however, a recent (2003 to 2010) study estimated the incidence to be about 2% [9]. This indicates that the incidence of CSE is increasing. In addition to pain and cosmetic problems, malignant transformation to clear cell carcinoma occurs in 1% of cases [10]; therefore, it is important for patients to be diagnosed and treated.

Although many theories have been postulated as to the cause of CSE, the most accepted theory is direct iatrogenic implantation of the endometrium into the wound margin during abdominal or pelvic surgery [11, 12].

Only approximately 20% of all patients with CSE show classic endometriosis symptoms of menstrual pain and accompanying changes in tumor size [11]. More often, patients complain of the presence of a mass (96%) and tenderness on palpation (87%) [1]. Differential diagnoses for these symptoms include fibromas, lipomas, suture granulomas, hernias, hematomas, lymphomas, desmoid tumors, and sarcomas. Various imaging modalities such as ultrasonography, computed tomography, and MRI are nonspecific but can help determine the extent of the disease and assist in planning for surgical resection [13]. While needle biopsies are useful for definitive diagnosis, it should be noted that they may result in the implantation of new endometrial tissue [14].

Appropriate surgical treatment offers the best opportunity to make a definitive diagnosis and treat CSE. Resection should include a tumor-free margin of at least 1 cm around the solid tissue [15]. In addition, endometriosis infiltrating the muscles of the abdominal wall requires en bloc resection of the underlying musculature and fascia. In our patient, the abdominal wall was reconstructed with an artificial mesh to restore the integrity of the abdominal wall and prevent postoperative hernia formation. Our report, in which the patient progressed without postoperative complications or recurrence, supports several previous reports that claim that appropriate resection of CSE and reconstruction with artificial mesh is useful [1, 6, 7]. Although the use of artificial mesh for abdominal wall repair can lead to complicated wound infections and risk of mesh erosion [16], it is often chosen as it is associated with a shorter surgical time and more stable results than skin and muscle flap surgery, which is more invasive.

Hormonal treatments such as gonadotropin-releasing hormone agonists, danazol, and progesterone have been used and provide only temporary relief, and symptoms often recur with discontinuation [6]. However, preoperative hormone therapy has been reported to be effective in reducing the size of the mass [17], and its use may be considered for future resection.

Our case demonstrates that accurate diagnosis of CSE, complete resection, and reconstruction with artificial mesh, can lead to good treatment outcomes and patient satisfaction. However, the long-term postoperative results cannot be determined at this time and require careful follow-up in the future.

Given the rarity of CSE, its diagnosis may be delayed, leading to frustration for both patients and physicians. CSE should be a differential considered by all surgeons. Proper mass resection and reconstruction of the abdominal wall where necessary are important for complete diagnosis and treatment.

## Data Availability

Data sharing is not applicable to this article as no datasets were generated or analyzed during the current study.
